# Bariatric Surgery Impact on Women with Polycystic Ovary Syndrome: A Prospective Cohort Study

**DOI:** 10.34133/research.0664

**Published:** 2025-04-09

**Authors:** Shumin Li, Xin Huang, Han Zhao, Shaozhuang Liu, Shigang Zhao

**Affiliations:** ^1^ State Key Laboratory of Reproductive Medicine and Offspring Health, Center for Reproductive Medicine, Institute of Women, Children and Reproductive Health, Shandong University, Jinan 250012, China.; ^2^ Division of Bariatric and Metabolic Surgery, Department of General Surgery, Qilu Hospital of Shandong University, Jinan, China.

## Abstract

Bariatric surgery has emerged as a promising intervention for obese women with polycystic ovary syndrome (PCOS), a condition strongly associated with obesity and anovulatory infertility. While weight management is a key therapeutic strategy, the optimal approach remains uncertain. A recent randomized controlled trial evaluated the impact of bariatric surgery on ovulation rates in obese women with PCOS. However, methodological limitations, including baseline body mass index discrepancies and minimal weight loss in the medical management group, necessitate cautious interpretation of the findings. To further investigate this issue, we conducted a prospective cohort study involving 192 women with PCOS who had undergone bariatric surgery. We assessed 30 reproductive and metabolic parameters at baseline and at 3, 6, and 12 months postoperatively. Most metabolic parameters improved significantly by 3 months postsurgery but plateaued thereafter, with only triglycerides and high-density lipoprotein cholesterol showing continued improvement. Reproductive outcomes demonstrated sustained improvements in ovulatory dysfunction, coinciding with a reduction in luteinizing hormone levels. However, testosterone levels and polycystic ovarian morphology showed limited improvement, while anti-Müllerian hormone levels remained unchanged. Despite the total weight loss exceeding 30%, further weight reduction did not proportionally enhance outcomes. These findings suggest that while bariatric surgery effectively improves metabolic health and ovulation in PCOS, its long-term hormonal and ovarian effects remain unclear and require further investigation. Head-to-head comparisons with emerging therapies are also urgently needed to refine weight management strategies for this high-risk population.

## Bariatric Surgery Impact on Women with Polycystic Ovary Syndrome

Polycystic ovary syndrome (PCOS) is the leading cause of anovulatory infertility, with 60% to 70% of affected individuals commonly experiencing obesity [1]. As the prevalence of overnutrition increases in society [2,3], the incidence of PCOS has been rising annually. Numerous Mendelian randomization studies have identified body mass index (BMI) as the causal factor in the development of PCOS [4]. Therefore, underscoring the importance of weight management is an effective strategy for its treatment. However, the optimal method for weight reduction, whether through diet, medication, or surgery, remains inconclusive, especially in terms of improving reproductive outcomes.

Bariatric surgery has emerged as a therapeutic option for obese women with PCOS; however, research in this area remains limited. Samarasinghe et al. [5] conducted a randomized controlled trial (RCT) to systematically compare the impact of bariatric surgery on ovulation rates in women with morbidly obese PCOS. While this pioneering study addressed a critical gap in the field, its findings should be interpreted with caution for several reasons. First, the baseline BMI in the medical group was notably lower than that of the surgical group, which might have influenced the outcomes. Second, the minimal weight loss observed in the medical group limits the comparability of weight reduction effects between the 2 groups. Third, despite the absence of marked weight loss in the medical group, the improvement in ovulatory dysfunction reached nearly 50% of that observed in the surgical group, raising questions about other potential contributing factors.

While bariatric surgery shows promising effects on the metabolic characteristics of PCOS, its impact on reproductive function must be interpreted more cautiously. The findings suggest that greater weight loss does not necessarily correlate with better reproductive outcomes, as evidenced by continuous decreases in anti-Müllerian hormone (AMH) and testosterone. Meanwhile, androstenedione, dehydroepiandrosterone sulfate, and hyperandrogenism scores (hirsutism, acne, and androgenic alopecia) did not show sustained declines over time. Additionally, the lack of relevant data on polycystic ovarian morphology (PCOM) in this RCT is noteworthy.

Conducting an RCT with such comprehensive indicators represents a notable advancement. Here, we share our prospective cohort follow-up data to critically evaluate the issue of bariatric surgery in PCOS. Our cohort consists of 192 PCOS patients diagnosed according to the Rotterdam criteria [6], all of whom underwent bariatric surgery. We collected data on anthropometric, metabolic, and reproductive indicators at baseline and at 3, 6, and 12 months postoperatively, along with the incidence of complications (Table).

**Table.** Clinical characteristics of obese women with PCOS preoperatively and at 3, 6, and 12 months postoperatively. Descriptive data are presented as mean (SD), median (IQR), or *n*/*N* (%).

Parameters	Preoperative, *n* = 192	3 M postoperative, *n* = 100	6 M postoperative, *n* = 92	12 M postoperative, *n* = 46	Overall *P*
Clinical phenotypes or diseases					
Oligo-ovulation ^a^	171/187 (91.4%) ^b^	37/90 (41.1%) ^b^	16/84 (19.0%) ^b^	2/43 (4.7%) ^b^	<0.001
Polycystic ovarian morphology ^c^	172/180 (95.6%) ^b^	52/70 (74.3%)	50/70(71.4%)	24/38 (63.2%)	<0.001
Hyperandrogenism ^d^	100/189 (52.9%) ^b^	21/93 (22.6%) ^e^^,^^f^	9/87 (10.3%) ^e^^,^^g^	6/44 (13.6%) ^e^	<0.001
Central obesity ^h^	167/185 (90.3%) ^b^	41/60 (68.3%) ^e^	38/64 (59.4%) ^e^	15/32 (46.9%) ^e^	<0.001
Diabetes ^i^	47/189 (24.5%) ^b^	0/96 (0.0%) ^e^	1/86 (1.1%) ^e^	0/44 (0.0%) ^e^	<0.001
Dyslipidemia ^j^	134/188 (71.3%) ^f^^,^^g^	59/94 (62.8%) ^k^	40/83 (48.2%) ^e^	14/44 (31.8%) ^e^^,^^g^	<0.001
Hypertriglyceridemia ^l^	76/189 (40.2%) ^b^	16/94 (17.0%) ^e^^,^^f^	5/86 (5.8%) ^e^^,^^g^	5/44 (11.4%) ^e^	<0.001
Hypercholesterolemia ^m^	66/189 (34.9%)	32/94 (34.0%)	25/87 (28.7%)	13/44 (29.5%)	0.726
Hyper-LDL-cholesterolemia ^n^	68/188 (36.2%)	32/95 (33.7%)	30/84 (35.7%)	10/44 (22.7%)	0.392
Hypo-HDL-cholesterolemia ^o^	58/189 (30.7%) ^f^^,^^k^	27/95 (28.4%) ^f^^,^^k^	9/85 (10.6%) ^b^	0/44 (0.0%) ^b^	<0.001
Metabolic parameters					
Weight loss (kg)	NA	24.83 (6.73) ^f^^,^^k^	33.99 (9.96) ^g^	38.34 (13.84) ^g^	<0.001
Percentage of total weight loss (%) ^p^	NA	22.05 (4.63) ^f^^,^^k^	29.78 (5.64) ^g^^,^^k^	34.23 (8.65) ^f^^,^^g^	<0.001
Percentage of excess weight loss (%) ^q^	NA	63.61 (28.39) ^f^^,^^k^	82.97 (23.06) ^g^^,^^k^	95.21 (28.36) ^f^^,^^g^	<0.001
Age (years)	29.00 (25.00–33.00)	29.00 (25.00–34.00)	30.00 (25.00–33.00)	28.00 (25.00–32.00)	0.966
Weight (kg)	110.00 (97.40–125.00) ^b^	85.30 (77.20–97.16) ^b^	75.00 (69.00–86.85) ^e^^,^^g^	69.50 (64.97–77.00) ^e^^,^^g^	<0.001
BMI (kg/m^2^)	40.60 (36.32–44.64) ^b^	31.68 (28.23–35.29) ^b^	28.26 (25.53–30.86) ^e^^,^^g^	25.73 (23.88–27.34) ^e^^,^^g^	<0.001
Waist-to-hip ratio	0.93 (0.88–0.98) ^b^	0.88 (0.85–0.92)	0.88 (0.83–0.94)	0.85 (0.82–0.91)	<0.001
Fat ratio (%)	43.70 (40.10–46.40) ^f^^,^^k^	41.50 (38.72–44.20) ^k^	37.80 (34.20–42.50) ^e^	34.40 (30.70–37.55) ^e^^,^^g^	<0.001
Lean mass ratio (%)	51.94 (49.14–55.53) ^b^	56.34 (52.69–58.56) ^e^^,^^k^	58.82 (55.28–62.34) ^e^	63.22 (57.75–66.65) ^e^^,^^g^	<0.001
Fasting glucose (mmol/l)	5.09 (4.64–5.78) ^b^	4.84 (4.54–5.17) ^e^	4.76 (4.58–5.04) ^e^	4.80 (4.54–4.93) ^e^	<0.001
Fasting C-peptide (ng/ml)	2.78 (2.13–4.03) ^b^	1.18 (0.82–1.71) ^e^	1.00 (0.71–1.58) ^e^	0.82 (0.70–1.03) ^e^	<0.001
Fasting insulin (μIU/ml)	25.81 (16.54–38.00) ^b^	10.68 (7.34–15.55) ^e^	9.01 (5.68–14.39) ^e^	11.82 (6.14–14.11) ^e^	<0.001
Glycated hemoglobin (%)	5.80 (5.50–6.32) ^b^	5.10 (4.90–5.30) ^e^	5.20 (5.00–5.30) ^e^	5.10 (4.90–5.40) ^e^	<0.001
Triglyceride (mmol/l)	1.55 (1.15–2.16) ^b^	1.17 (0.95–1.49) ^b^	0.92 (0.74–1.19) ^e^^,^^g^	0.86 (0.64–1.11) ^e^^,^^g^	<0.001
Cholesterol (mmol/l)	4.79 (4.28–5.47)	4.69 (4.21–5.38)	4.76 (4.11–5.28)	4.70 (4.40–5.29)	0.6
LDL-C (mmol/l)	3.11 (2.70–3.58)	3.05 (2.65–3.55)	3.08 (2.45–3.70)	2.96 (2.56–3.30)	0.738
HDL-C (mmol/l)	1.08 (0.96–1.20) ^f^^,^^k^	1.10 (0.98–1.25) ^f^^,^^k^	1.24 (1.08–1.42) ^b^	1.42 (1.32–1.60) ^b^	<0.001
Non-HDL-C (mmol/l)	3.71 (3.24–4.30) ^f^^,^^k^	3.61 (3.07–4.18)	3.36 (2.84–4.07) ^e^	3.20 (2.90–3.77) ^e^	0.014
Nonesterified fatty acid (μmol/dl)	65.00 (55.70–82.00) ^f^^,^^k^	64.80 (52.92–79.75) ^k^	55.65 (42.45–65.75) ^e^	41.00 (34.05–58.65) ^e^^,^^g^	<0.001
HOMA-IR ^r^	5.75 (3.76–9.81) ^b^	2.26 (1.49–3.41) ^e^	1.84 (1.29–2.91) ^e^	1.67 (1.17–2.98) ^e^	0.009
Adipo-IR ^s^	99.11 (62.71–155.72) ^b^	36.71 (28.86–55.12) ^e^	30.57 (19.18–61.59) ^e^	27.37 (19.57–37.80) ^e^	<0.001
TyG index^t^	8.78 (8.44–9.18) ^b^	8.40 (8.19–8.69) ^b^	8.16 (7.94–8.44) ^e^^,^^g^	8.03 (7.77–8.46) ^e^^,^^g^	<0.001
Reproductive parameters					
Testosterone (nmol/l)	1.72 (1.18–2.19) ^b^	1.06 (0.78–1.53) ^e^	0.88 (0.57–1.27) ^e^	1.03 (0.75–1.26) ^e^	<0.001
Anti-Müllerian hormone (ng/ml)	3.70 (2.56–5.42)	4.21 (2.88–5.61)	3.49 (2.12–5.15)	3.83 (1.95–5.22)	0.475
Follicle-stimulating hormone (mIU/ml)	5.54 (4.61–6.39)	5.04 (3.90–6.27)	4.87 (3.74–6.54)	5.43 (4.32–6.39)	0.22
Luteinizing hormone (mIU/ml)	8.13 (6.57–10.69) ^b^	6.33 (4.24–9.49) ^e^	6.14 (3.78–9.45) ^e^	5.95 (3.81–8.34) ^e^	0.069
LH/FSH	1.56 (1.21–2.04)	1.44 (0.90–2.16)	1.16 (0.75–1.88)	1.29 (0.81–1.91)	0.626
Estradiol (pmol/l)	174.40 (137.25–230.22) ^g^	123.00 (62.80–211.80)	152.30 (51.50–281.75)	186.95 (125.40–322.65)	0.538
Progesterone (nmol/l)	0.58 (0.36–0.94) ^k^	0.50 (0.19–6.60)	0.67 (0.20–4.42)	0.92 (0.40–5.70)	<0.001
Prolactin (μIU/ml)	304.75 (244.00–466.30)	287.79 (210.25–384.57)	267.75 (204.27–406.13)	296.45 (219.75–397.37)	0.191

PCOS, polycystic ovary syndrome; IQR, interquartile range; NA, not applicable; LH, luteinizing hormone; FSH, follicle-stimulating hormone; LDL-C, low-density lipoprotein cholesterol; HDL-C, high-density lipoprotein cholesterol; HOMA-IR, homoeostatic model assessment for insulin resistance; Adipo-IR, adipose tissue insulin resistance; TyG, triglyceride-glucose

^a^ Oligo-ovulation is characterized by a cycle length of fewer than 21 d or more than 35 d, fewer than 8 cycles in the past 12 months, or absence of menstruation.

^b^ *P* < 0.05 compared with any other 3 groups.

^c^ Polycystic ovarian morphology is defined as the presence of 12 or more follicles measuring 2 to 9 mm in diameter in each ovary and/or an ovarian volume of more than 10 ml as determined by ultrasound.

^d^ Hyperandrogenism is defined as serum total testosterone more than 1.67 nmol/l.

^e^ *P* < 0.05 compared with preoperative.

^f^ *P* < 0.05 compared with 6 M (6 months) postoperative.

^g^ *P* < 0.05 compared with 3 M (3 months) postoperative.

^h^ Central obesity is defined as a waist circumference greater than 85 cm.

^i^ Diabetes is defined as a fasting glucose more than 7.0 mmol/l or glycated hemoglobin more than 6.5%.

^j^ Dyslipidemia is defined as the presence of at least one of the following: hypertriglyceridemia, hypercholesterolemia, hyper-LDL-cholesterolemia, or hypo-HDL-cholesterolemia.

^k^ *P* < 0.05 compared with 12 M (12 months) postoperative.

^l^ Hypertriglyceridemia is defined as triglyceride more than 1.7 mmol/l.

^m^ Hypercholesterolemia is defined as cholesterol more than 5.2 mmol/l.

^n^ Hyper-LDL-cholesterolemia is defined as LDL more than 3.35 mmol/l.

^o^ Hypo-HDL-cholesterolemia is defined as HDL less than 1 mmol/l.

^p^ Percentage of total weight loss = BMI loss/(BMI-0) × 100.

^q^ Percentage of excess weight loss = BMI loss/(BMI-25) × 100.

^r^ HOMA-IR = fasting glucose (mmol/l) × fasting insulin (μIU/ml)/22.5 [5].

^s^ Adipo-IR = nonesterified fatty acid (mmol/l) × fasting insulin (pmol/l) [9].

^t^ TyG = ln(triglyceride (mg/dl) × fasting glucose (mg/dl)/2) [10].

Significant weight loss was observed consistently across all postoperative time points, as evidenced by substantial reductions in BMI, fat ratio, and related anthropometric indicators or disease status like central obesity, reflecting the effectiveness of the intervention. The average values of several glycometabolic and lipid-related indices, including homoeostatic model assessment for insulin resistance, adipose tissue insulin resistance, triglyceride-glucose index, fasting C-peptide, low-density lipoprotein cholesterol, high-density lipoprotein cholesterol (HDL-C), non-HDL-C, and nonesterified fatty acids, continued to decrease over a year postoperative. However, it is noteworthy that, statistically, most of these metabolic indices showed significant changes by 3 months postsurgery, after which they stabilized. Interestingly, among these indices, those that continued to show significant decreases or increases over time included triglycerides and HDL-C levels.

Among the characteristics of PCOS, ovulatory dysfunction showed significant and sustained improvement postoperatively, likely driven by a continuous reduction in luteinizing hormone levels. While hyperandrogenemia and testosterone levels improved initially, they appeared to plateau or even slightly rebound with longer follow-up. For PCOM, surgical improvement was challenging, with 63.2% of PCOS patients still exhibiting PCOM at 12 months postoperative, suggesting that the effects of weight loss surgery on follicle development may be complex. Recent PCOS guidelines suggest using AMH as a substitute for PCOM [7]; however, we also found no sustained decline in AMH, contrasting with Samarasinghe et al.’s findings. This indicates the need for longer follow-up, especially regarding the impact of surgery on ovarian function. Moreover, weight loss and the associated reductions in insulin resistance and hyperinsulinemia following bariatric surgery can enhance folliculogenesis and granulosa cell function, potentially influencing AMH production. Notably, studies have demonstrated that for each unit increase in BMI, AMH levels decrease by an average of −0.015 to −0.2 ng/ml, suggesting a strong inverse relationship between BMI and AMH [8]. This may partly explain the absence of a significant AMH decline in our cohort, as weight loss appears to mitigate the inhibitory effect of obesity on AMH production. In summary, further long-term follow-up and mechanistic studies are warranted to fully elucidate the impact of weight loss surgery on ovarian function.

However, it is also worth noting that the alteration of PCOM is challenging. Our longitudinal observation, based on multi-time-point follow-up data, indicates that while the degree of weight reduction becomes increasingly pronounced postoperatively, with total weight loss reaching over 30%, many reproductive and metabolic indicators tend to plateau. This suggests that further weight loss may not necessarily yield additional benefits. Meanwhile, rapid weight loss may induce temporary hormonal fluctuations, such as changes in luteinizing hormone pulse frequency, and can temporarily disrupt the hypothalamic–pituitary–ovarian (HPO) axis, potentially masking long-term improvements in reproductive function. For instance, rapid reductions in adipose tissue may transiently alter leptin and adipokine levels, which play roles in regulating gonadotropin secretion and ovarian activity. However, it is important to note that these fluctuations are likely short-term, and the HPO axis can gradually stabilize. In our cohort, improvements in ovulation and menstrual regularity were observed as early as 3 months postsurgery, with stabilization at 6 to 12 months, suggesting that long-term hormonal equilibrium may eventually dominate. We acknowledge that the current 12-month follow-up period limits our ability to fully capture these dynamics. Future studies with extended follow-up (e.g., 3 to 5 years) are needed to clarify whether the observed plateau represents a transient phase or a ceiling effect of surgical intervention. In the absence of more clinical trial and long-term follow-up data, it is prudent to advocate for a moderate weight reduction strategy as a more responsible approach to patient care.

In the future, well-designed clinical studies, including RCTs and cohort studies, are essential to elucidating the optimal role of bariatric surgery for PCOS patients with obesity from multiple perspectives. Future RCTs should focus on comparing bariatric surgery with nonsurgical interventions, including emerging therapies such as glucagon-like peptide 1 receptor agonists (e.g., semaglutide and tirzepatide), as well as diet and exercise programs, to evaluate their effects on reproductive, metabolic, and psychological outcomes in PCOS patients. Although weight loss is highly effective, it is crucial for these studies to also determine the optimal range of weight reduction that leads to synergistic improvements in the reproductive and metabolic profiles of obese patients with PCOS. Importantly, excessive weight loss may inflict considerable damage on the HPO axis. Given that current follow-up studies on bariatric surgery for PCOS patients are limited to 1 year, extending follow-up periods to 5 years or more is crucial. Long follow-up will help to assess the long-term efficacy and safety of bariatric surgery, including sustained weight loss, metabolic and reproductive stability, and surgical complications. Moreover, from a reproductive standpoint, future investigations should more thoroughly evaluate the impact of bariatric surgery on fertility rates, pregnancy complications, and offspring health, particularly since many PCOS patients pursue rapid weight loss primarily to enhance fertility. Given the chronic nature of PCOS, monitoring these outcomes over extended periods will be crucial for fully understanding the benefits and risks. We believe that such studies will provide critical insights into optimal treatment strategies for PCOS patients and help guide clinical decision-making.

## Study Design and Patients

The study enrolled patients meeting the following inclusion criteria: (a) a BMI of ≥32.5 kg/m^2^ or a BMI of ≥27.5 kg/m^2^ accompanied by obesity-related comorbidities or at least 2 components of metabolic syndrome; (b) age between 18 and 42 years; and (c) a diagnosis of PCOS based on the 2003 Rotterdam criteria, defined by the presence of 2 or more of the following features: oligo- or amenorrhea, clinical or biochemical signs of hyperandrogenism, and PCOM on ultrasound. Only patients with complete clinical data for these 3 key features were included in the follow-up analysis. Exclusion criteria for bariatric surgery for obese PCOS included uncontrolled psychiatric disorders, substance abuse, unrealistic expectations, poor compliance, inability to tolerate surgery, or plans for pregnancy within postoperative 1 year.

## Ethical Statement

We conducted a prospective cohort, which was approved by the Medical Ethics Committee of Shandong University Qilu Hospital (No. 2019-135) and registered at the Chinese Clinical Trial Registry (No. ChiCTR1900026845), to investigate the effects of bariatric surgery on obese women with PCOS.

## Statistical Analysis

All statistical analyses were performed using R version (4.3.1). For comparing PCOS patients at baseline and at 3, 6, and 12 months postoperatively, we used the Mann–Whitney *U* test (Wilcoxon rank-sum test) for pairwise comparisons, and we applied the Bonferroni correction to adjust the *P* values for multiple testing correction.
